# A randomized trial of permanent supportive housing for chronically homeless persons with high use of publicly funded services

**DOI:** 10.1111/1475-6773.13553

**Published:** 2020-09-25

**Authors:** Maria C. Raven, Matthew J. Niedzwiecki, Margot Kushel

**Affiliations:** ^1^ Department of Emergency Medicine University of California, San Francisco San Francisco CA USA; ^2^ Philip R. Lee Institute for Health Policy Studies University of California, San Francisco San Francisco CA USA; ^3^ Mathematica Policy Research Oakland CA USA; ^4^ Center for Vulnerable Populations University of California, San Francisco San Francisco CA USA; ^5^ Department of Medicine University of California, San Francisco San Francisco CA USA; ^6^ UCSF Benioff Homelessness and Housing Initiative San Francisco CA USA

**Keywords:** criminal justice, frequent users, homelessness, integrated data, permanent supportive housing

## Abstract

**Objective:**

To examine whether randomization to permanent supportive housing (PSH) versus usual care reduces the use of acute health care and other services among chronically homeless high users of county‐funded services.

**Data Sources:**

Between 2015 and 2019, we assessed service use from Santa Clara County, CA, administrative claims data for all county‐funded health care, jail and shelter, and mortality.

**Study Design:**

We conducted a randomized controlled trial among chronically homeless high users of multiple systems. We compared postrandomization outcomes from county‐funded systems using multivariate regression analysis.

**Data Collection:**

We extracted encounter data from an integrated database capturing health care at county‐funded facilities, shelter and jails, county housing placement, and death certificates.

**Principal Findings:**

We enrolled 423 participants (199 intervention; 224 control). Eighty‐six percent of those randomized to PSH received housing compared with 36 percent in usual care. On average, the 169 individuals housed by the PSH intervention have remained housed for 28.8 months (92.9 percent of the study follow‐up period). Intervention group members had lower rates of psychiatric ED visits IRR 0.62; 95% CI [0.43, 0.91] and shelter days IRR 0.30; 95% CI [0.17, 0.53], and higher rates of ambulatory mental health services use IRR 1.84; 95% CI [1.43, 2.37] compared to controls. We found no differences in total ED or inpatient use, or jail. Seventy (37 treatment; 33 control) participants died.

**Conclusions:**

The intervention placed and retained frequent user, chronically homeless individuals in housing. It decreased psychiatric ED visits and shelter use, and increased outpatient mental health care, but not medical ED visits or hospitalizations. Limitations included more than one‐third of usual care participants received another form of subsidized housing, potentially biasing results to the null, and loss of power due to high death rates. PSH can house high‐risk individuals and reduce emergent psychiatric services and shelter use. Reductions in hospitalizations may be more difficult to realize.

**What This Study Adds**

- We found that the PSH program intervention was able to house 86 percent of chronically homeless adults randomized to the treatment group based on their high use of multiple systems who were randomized to the treatment group.
- On average, it took 2.5 months for participants randomized to housing to become housed and 70 percent moved at least once, demonstrating that PSH can be successful with high‐risk participants but requires time and flexibility.
- By using a randomized controlled trial design, we found that those randomized to housing (versus usual care) had lower use of psychiatric emergency departments and shelters, but did not have large reductions in service use described in previous uncontrolled studies.

## 1 INTRODUCTION

Homelessness is associated with high use of acute health care services, including emergency department (ED)^1^, ^2^ and inpatient care.^3^ Among homeless individuals, a small group (referred to as “frequent users”) account for a large proportion of all acute service use.^4^ Most of these frequent users are chronically homeless individuals with tri‐morbid chronic physical and mental health conditions and substance use disorders.^5^, ^6^, ^7^ In addition to high rates of use of ED and inpatient care, many have high use of other publicly funded services (jail, homeless shelters)^8^, ^9^ and low use of longitudinal, outpatient health care. Interventions to maintain housing and reduce acute care service use in this population are a key policy interest for payers and providers.

Permanent supportive housing (PSH), defined as subsidized housing with closely linked, voluntary supportive services (eg, case management, physical and mental health services, substance use treatment services) provides permanent housing for people with chronic homelessness and behavioral health conditions.^10^ PSH is offered on a “housing first”^11^ basis, meaning clients are not required to be sober or engage in treatment. Most of the literature evaluating the effect of PSH on health care and other service utilization has used pre–post, noncontrolled, study designs.^12^, ^13^ While these have suggested large reductions in service use, they face threats to internal validity. By including only people who have enrolled successfully in PSH, these studies do not provide insights into reach.^14^, ^15^ Because they focus on change in utilization of a group selected on the basis of high use, they are susceptible to regression to the mean.

Santa Clara, CA created 112 units of PSH earmarked for high users of multiple public systems of care; over time, the project increased to 130 units. As the population who met criteria greatly exceeded PSH supply, we utilized lottery conditions to conduct a randomized controlled trial to examine the reach and effect on service use of PSH comparing those randomized to PSH versus usual care.

## 2 METHODS

We evaluated differences in use of county health, shelter and criminal justice services, housing placement and maintenance, and mortality, comparing individuals randomized to PSH to usual care. We used an intention‐to‐treat framework. Members of the control group were eligible for PSH provided through other county‐funded programs.

We evaluated Project Welcome Home, a “Pay for Success” based project to create PSH for the highest users of county public systems (ED, inpatient services, and jail) in Santa Clara, CA. Approximately 28 percent of units were scattered site and 72 percent were congregated. Most of the congregate units are set‐aside units in private or nonprofit affordable housing buildings. A limited number of other units are located in converted hotels owned by local housing providers. Housing and case management services were provided by Abode Services. Between July 2015 and September 2019, we assessed service use from the Santa Clara County (SCC) administrative claims data for all county‐funded health care, criminal justice, and shelter services and assessed deaths from county death certificates for all study participants. The project is ongoing.

### 2.1 Study screening and enrollment

The screening process includes administrative data screening to determine eligibility by usage criteria, followed by an in‐person screening to determine other eligibility criteria and ability to consent. Randomization occured after consent. A proprietary platform integrates study data with real‐time data feeds from multiple sources.

Staff screened potential participants based on their use of county‐funded services over the prior 1‐2 years. Our research team developed an electronic triage tool that uses administrative data to predict the likelihood of future high use of county‐funded services. To meet criteria, potential participants must have used various combinations of the ED and psychiatric ED, medical and psychiatric inpatient stays in the County‐funded public hospital, and/or jail over the past 1‐2 years, at high enough levels to meet a threshold score. We embedded the triage tool into the study database and generated a list of potentially eligible participants with the highest scores, redoing the calculation throughout the enrollment period. All county agencies or service providers could refer individuals they suspected met eligibility criteria, but study staff always used the list generated by the triage tool to confirm initial eligibility. County staff used this list to outreach to the highest using individuals.

In addition to meeting threshold use levels, participants had to: (a) meet the Federal definition of chronic homelessness (homeless for more than a year or 4 or more episodes in the prior three years that last for more than a year total, with a disabling condition); (b) live in SCC; (c) not be incarcerated; (d) not engage in another intensive case management program or other permanent supportive housing program; (e) not require nursing home level care; and (f) not have metastatic cancer or qualify for hospice care.

After they identified that a prospective participant met eligibility requirements, the staff conducted informed consent, using a teach‐back method to ensure understanding. Then, staff randomized participants using a random number generator. Staff referred individuals randomized to the intervention group to Abode for engagement in the permanent supportive housing. They informed participants randomized to usual care that they remained eligible for all standard services, including other permanent supportive housing programs provided by the County. We continued enrollment until all the units filled and then enrolled additional participants whenever a unit opened, through participants’ leaving housing, requiring higher level of care, or death.

### 2.2 Intervention

If an individual agreed to engage with Abode, they began to deliver case management services, even if a housing unit was not yet available. If the individual did not agree to engage immediately, staff continued to reach out to build rapport at least one time per week for six to nine months (depending on program capacity). If the staff could not engage the participant, the staff ceased outreach attempts.

Abode's case management services use an Intensive Case Management^16^ model. This includes community‐based services, provided by master's level social behavioral health providers, bachelor's level case managers, and staff with lived experience (peers). Abode integrated these services with a flexible array of housing options delivered through a Housing First approach, to provide temporary housing (if no permanent unit available immediately), permanent supportive housing, and rehousing (locating new housing units if the participant was evicted or otherwise lost a unit). Participants received a rental subsidy to pay for the housing unit. Caseloads ranged from 1:10 to 1:15. Abode did not employ nurses or physicians.

Abode offers a range of additional supportive services to participants. These include mental health and substance use services; medication support, community living skills, educational and vocational support, money management, leisure and spiritual opportunities, and connection to primary care. Those in the intervention group who were not lost to follow‐up continued to receive case management services as part of the PSH intervention throughout the intervention, whether or not they remain housed.

### 2.3 Usual care

At the time of enrollment, staff provided all participants randomized to usual care referrals to shelters and other homeless services. These participants remained eligible to receive all services provided for individuals experiencing homelessness in SCC, including any form of shelter, and temporary or permanent housing, including PSH not designated for Project Welcome Home. Staff conducted a Vulnerability Index‐Service Prioritization Decision Assistance Tool (VI‐SPDAT)^17^ assessment, in order to place clients on a list for County housing interventions. During the intervention period, SCC created other programs to provide PSH to chronically homeless individuals, and participants in the control group were eligible to receive case management services through other county programs.

### 2.4 Data

We extracted encounter data from the SCC integrated database that combines county‐funded health care utilization data (ED and inpatient stays for medical or psychiatric causes, outpatient mental health and substance use treatment, outpatient medical treatment) with data from the County jail (all jail utilization) and shelter data from the Homeless Management Information System.

We linked data using participants’ social security numbers, names, and dates of birth. An outside entity linked data via an entity resolution process that used name, date of birth, social security number, and unique identifiers within each system (such as medical record numbers) coupled with a process to review and update any matches across systems.

### 2.5 Participant characteristics

We defined age at the date of enrollment. We included self‐reported sex, Hispanic ethnicity, race (White, Black, other), smoking status (current versus former/never), insurance status (Medicaid, Medicare, or both). We report on service use characteristics for county‐funded services in the two years prior to enrollment.

#### 2.5.1 Health services utilization

Santa Clara Valley Medical Center (SCVMC), a public safety net hospital is the main acute care service provider for homeless patients in SCC. SCVMC was the source of data for primary care use, ED visits (including psychiatric ED), and inpatient hospitalizations (including psychiatric admissions). Hospitals that contract with SCVMC to provide psychiatric inpatient care provided data. We did not have access to data on physical health visits from other settings.

SCVMC provided data on outpatient mental health care and substance use treatment, including initial and ongoing, group and individual treatment, including mental health outpatient services provided by Abode.

We examined whether participants identified a regular source of non‐ED outpatient care. We examined the number of primary care physician (PCP) visits each year, as recorded in County administrative data. We defined a PCP visit as a visit to a physician, nurse practitioner, or physicians’ assistant at a primary care clinic. We categorized physical health ED visits in three ways: visits that result in discharge, visits that result in hospital admission, and total visits. We defined inpatient hospital care as the number of hospital admissions a participant had at SCVMC. We examined the number of acute bed days (length of stay). To examine mental health services use, we examined a participant's number of outpatient mental health appointments at county facilities, number of visits to the county psychiatric ED that resulted in discharge, and number of psychiatric hospitalizations. Regarding substance use treatment services use, we examined participants’ number of days in inpatient and outpatient detoxification and rehabilitation facilities, as well as other outpatient clinical substance use services.

#### 2.5.2 Criminal justice

The County provided jail data through the Criminal Justice Information System for study participants that included the timing of arrests and the length of stay in SCC jails.

#### 2.5.3 Housing and shelter outcomes

For all participants, we report on whether they received housing at any point during the study. For participants in the intervention group, Abode reported whether and when they obtained, left, and regained housing. For descriptive purposes, we examined how long (after study enrollment) it took for Abode to house each participant and how many times participants needed to be rehoused. To assess housing retention for those in the intervention group housed by Abode, we examined the ratio between total days each participant remained housed and the total possible housing days (the participant's first move‐in date until the end of the study follow‐up period). We converted our result to months.

For participants in the intervention group who did not receive housing by Abode and all participants in the usual care group, we obtained data from SCC that identified whether or not the participant had received housing through other County housing programs. If so, these data included the last recorded date of housing placement and whether or not the participant remained in housing or exited. For those who had been housed but had exited, the data included where they exited to (eg, another housing placement outside of the county; with family; to homelessness).

For all participants, we checked for any use of the emergency shelters in SCC through data from the SCC Homeless Management Information System. We calculated amount of time in shelter. We did not have data on privately funded shelters.

#### 2.5.4 Mortality

Abode provided data on death for all participants who died while living in Abode housing. We queried County death certificate data on all participants who did not appear in any source of study data for 6 or more months.

### 2.6 Data analysis

To assess outcomes, we grouped data into one‐year spans of time for each individual in the treatment and control group. For example, if an individual was enrolled for 4 years, they would have four separate one‐year spans in the regression analysis. The use of spans allows us to include the most available data for each individual in the study.

For participants who had potential spans that lasted ≥6 but <12 months, we prorated utilization counts. To account for outliers in the data, we top coded all span‐level counts to the 99th percentile. We included indicators in the regression analysis to signify the year in the program in order to account for patterns of use that may decrease or increase over time.

We censored spans at the time of death. To account for the possibility that participants moved out of County, we censored data 6 months after the last point of contact and constructed spans with the data that remained.

We used negative binomial regression analysis on count data outcomes using an intention‐to‐treat framework based on assignment to the treatment group. Since the treatment and control groups were balanced on baseline characteristics, we did not include covariates in the negative binomial regressions. We controlled for the time since enrollment (span indicators), to account for the differences in enrollment period. We present results as incidence rate ratios (IRR). We clustered standard errors at the individual level to account for individuals with multiple spans.

In sensitivity analyses, we recoded outcome variables to a binary indicator for whether an individual used any of a given service within the one‐year span. We conducted sensitivity analyses (Table S1). We explored allowing the treatment effect to vary by how long the individual was enrolled in the program by including interaction terms for treatment status and year indicators (results not reported).

## 3 RESULTS

### 3.1 Sample characteristics

**FIGURE 1:**
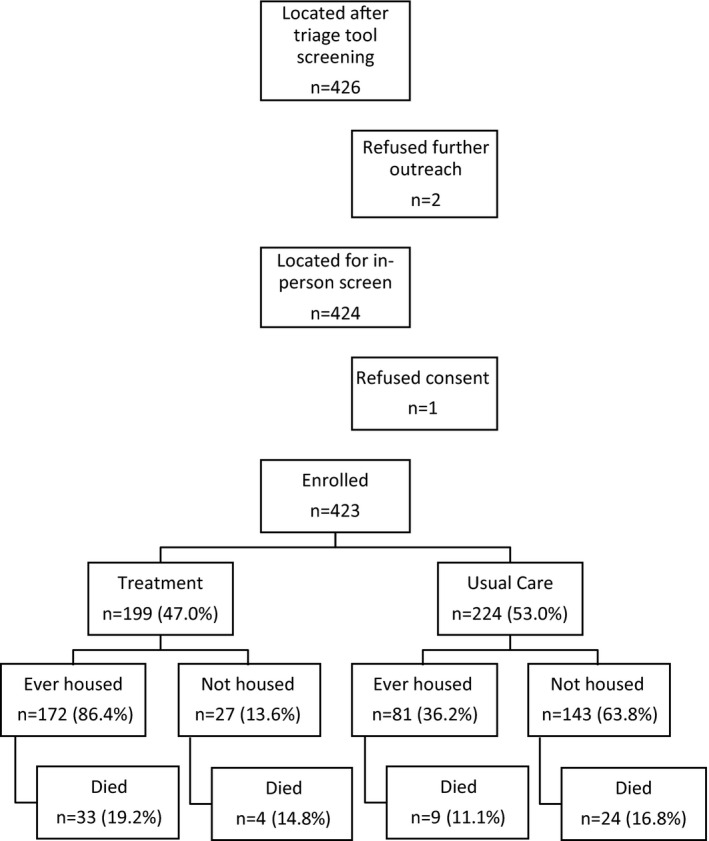
Study Enrollment with housing and mortality outcomes comparing PSH intervention group to usual care. Abbreviation: PSH, permanent supportive housing

After identification by the triage tool, county or health services staff approached 426 potential participants. Two refused further outreach. Study staff approached 424 participants, one of whom refused consent. We enrolled 423 participants: 199 in the PSH intervention group and 224 in usual care (Figure 1). We report on demographics’ and county‐funded services’ use in the two years prior to enrollment in Table 1. We found no meaningful differences in demographic characteristics between the groups. The participants’ mean age was 51.8 years for treatment, 51.2 years control. Most were male (72 percent intervention, 71 percent control). A quarter identified as Hispanic (24 percent intervention, 25 percent control). Two‐thirds identified as White (64 versus 66 percent) while a small proportion identified as Black (13 percent versus 15 percent). In the two years prior to enrollment, those in the treatment group averaged 5.1 inpatient admissions, 19.0 ED visits, 3.7 jail stays, and 36.7 shelter days. They had a mean of 6.5 outpatient substance use treatment visits and 26.0 outpatient mental health visits. The control group utilization was not statistically different from the treatment group. However, participants in the control group had a higher prevalence of reporting a regular source of care in the two years prior to enrollment (mean 83 percent vs. 70 percent).

**TABLE 1:** Study sample demographic characteristics and health services use in the two years prior to enrollment, treatment versus usual care

	Mean, Treatment	Mean, Control	Difference
*Follow‐up duration*			
Months	35.8	36.8	1.1
*Demographics*			
Male	72%	71%	−2%
Hispanic ethnicity	24%	25%	1%
White race	64%	66%	2%
Black race	13%	15%	3%
Other race	23%	19%	−4%
Age in years	51.8	51.2	−0.595
Currently Smoking	65%	66%	1%
*Insurance*			
Medicaid insurance	65%	66%	1%
Medicare insurance	73%	73%	−1%
*Health services use*			
Regular source of care (not ED)	70%	83%	14%
Ambulatory care visits	7.3	8.8	1.5
Inpatient psychiatry stays	0.2	0.3	0.1
Total inpatient stays	5.1	4.8	1.5
Total bed days	14.5	15.1	0.6
ED visits (total)	19.0	20.1	1.1
ED visits discharged home	16.7	18.0	1.4
ED visits admitted	2.3	2.1	−0.3
Emergency psychiatry visits	4.7	5.4	0.6
*Jail*			
Jail stays	3.7	2.8	−0.9
Jail days	56.0	61.9	5.9
*Shelter use*			
Shelter stays	30.8	37.5	6.6
Shelter days	36.7	42.0	5.2
*Outpatient behavioral health*			
Outpatient substance use treatment visits	6.5	5.5	−1.0
Outpatient mental health visits	26.0	28.9	2.9

Abbreviation: ED, emergency department.

### 3.2 Descriptive statistics—outcomes

During the follow‐up period, 86 percent of those randomized to the PSH intervention received housing compared with 36 percent of those in the control group (Table 2). Of the 199 people randomized to intervention, 169 received housing through this program; three received housing through another program. The average time from enrollment to housing placement was 74.2 days. Of the 169 participants housed by Abode, 119 (70.4 percent) moved at least once. Three‐quarters (72.0 percent) of the participants who required rehousing had no housing gap between placements. On average, housed intervention group participants moved an average of 2.06 times during the follow‐up period (range 1‐10 times). The 169 participants housed by Abode have remained housed for an average of 28.8 months and have been retained in housing (without gaps) for 92.9 percent of the possible study follow‐up period. When examining one‐year spans over the course of the study, the intervention group was housed in 84.4 percent of a given span compared to 20.1 percent of those in the control group. (*P* < .01). Individuals in the treatment group had 6.6 shelter days per year versus 16.8 in the control group (*P* < .01).

**TABLE 2:** Logistic and negative binomial regression analysis of treatment status on Medical, criminal justice, and housing outcomes

	Ever housed	ED visits	Emergency psych visits	Total inpatient stays	Inpatient psych stays	Jail stays	Shelter days	Outpatient substance use treatment visits	Outpatient mental health visits
Treatment Group	22.34**	0.85	0.62*	0.97	0.73	1.01	0.30**	0.76	1.84**
	[11.69,42.68]	[0.67,1.08]	[0.43,0.91]	[0.70,1.35]	[0.36,1.45]	[0.73,1.40]	[0.17,0.53]	[0.46,1.24]	[1.43,2.37]
Span 1 (reference)	1.0	1.0	1.0	1.0	1.0	1.0	1.0	1.0	1.0
	—	—	—	—	—	—	—	—	—
Span 2	1.11	0.81**	0.79*	0.80*	0.66	0.89	0.51**	0.65*	0.95
	[0.95,1.29]	[0.71,0.93]	[0.64,0.97]	[0.66,0.96]	[0.31,1.44]	[0.76,1.04]	[0.33,0.78]	[0.45,0.95]	[0.82,1.09]
Span 3	1.13	0.74**	0.80	0.70*	0.78	0.83	0.34**	0.18**	0.86
	[0.91,1.40]	[0.62,0.88]	[0.63,1.01]	[0.53,0.92]	[0.35,1.74]	[0.67,1.02]	[0.21,0.55]	[0.13,0.27]	[0.71,1.05]
Span 4	1.51*	0.63**	0.69*	0.51**	0.61	0.81	0.32**	0.16**	0.85
	[0.84,2.11]	[0.49,0.81]	[0.49,0.97]	[0.35,0.75]	[0.21,1.82]	[0.61,1.08]	[0.14,0.73]	[0.09,0.27]	[0.67,1.09]
N	1070	1070	1070	1070	1070	1070	1070	1070	1070

Note

All results presented as an odds ratio for the binary outcome “ever housed” from a logistic regression and as incidence rate ratios for all other outcomes from negative binomial regressions. 95% confidence intervals also presented. Covariates include treatment status as well as span indicators to control for the time since enrollment. No other covariates were included as the treatment was randomly assigned.

Abbreviation: ED, emergency department.

* *P* < .05, **P < .01.

Individuals in the treatment group received outpatient mental health treatment 37.3 times per year versus 19.7 times per year in the control (*P* < .01). Those in the treatment group had fewer psychiatric emergency visits per year as compared to the control group: 1.3 visits per year in the treatment group versus 1.9 per year in the control group (*P* < .01).

**FIGURE 2:**
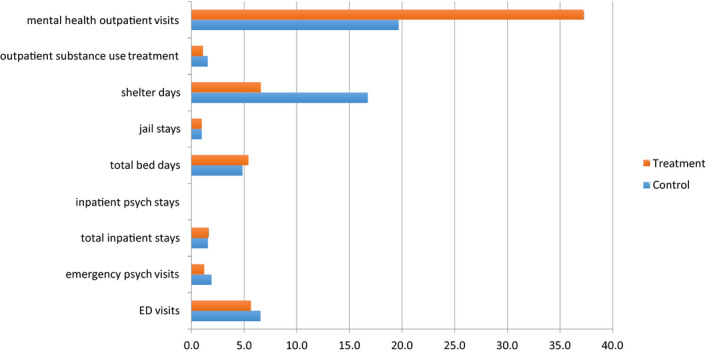
Outcome variables, PSH intervention versus usual care. Abbreviations: ED, emergency department; PSH, permanent supportive housing [Colour figure can be viewed at wileyonlinelibrary.com]

Intervention and control groups had similar levels of ED visits, inpatient admissions, psychiatric inpatient admissions, jail stays, and outpatient substance abuse treatment services. We present mean utilization rates during the study period for outcome variables included in the regression analysis in Figure 2.

A total of 37 (18.6 percent) in the intervention group and 33 (14.7 percent) in the control group died during the study follow‐up period. Of those who died, 89.2 percent of those in the intervention group had ever received housing during the study period, compared with 29.0 percent of those in the control group.

### 3.3 Regression analysis

The treatment group was more likely to be ever housed during the study period (odds ratio [OR]: 22.34, 95% CI: [11.69,42.68]). The intervention group had nearly two‐thirds fewer days in shelter compared to the control group (IRR: 0.30, 95% CI: [0.17, 0.53]). Individuals in the treatment group had nearly twice as many outpatient mental health visits as those in the control group (IRR: 1.84, 95% CI: [1.43,2.37]). Assignment to the treatment group was associated with a 38 percent reduction in psychiatric ED visits (IRR: 0.62, 95% CI: [0.43,0.91]).

No other differences were statistically significant. Those in the treatment group had 15 percent fewer ED visits (IRR: 0.85, 95% CI: [0.67,1.08]) and 27 percent fewer psychiatric inpatient admissions (IRR: 0.73, 95% CI: [0.36,1.45]) but the difference did not reach statistical significance. Both groups had similar rates of inpatient admissions (IRR: 0.97, 95% CI: [0.70,1.35]) and jail stays (IRR: 1.01, 95% CI: [0.73,1.40]). Those in the treatment group received 24 percent fewer outpatient substance use treatment visits (IRR: 0.76, 95% CI: [0.46,1.24]), but the result was not statistically significant. When we interacted treatment status with year of enrollment, we found no statistically significant differences in the treatment effect for any of the outcomes studied, although we were underpowered to do so. We examined differences in the number of hospital days and jail days as secondary outcomes and found no differences. (Appendix S1).

## 4 DISCUSSION

In a randomized control trial comparing chronically homeless individuals who were the highest users of multiple systems of care in Santa Clara, CA randomized to receive permanent supportive housing versus usual care, we found that participants randomized to PSH experienced reductions in psychiatric ED and shelter use but no reductions in use of medical EDs, hospitals, or jail. Despite the social complexity of the study participants, 86 percent of those randomized to PSH entered housing and remained in housing for the vast majority (92.9 percent) of the study follow‐up period.

We found a significant reduction in use of psychiatric emergency services and a concomitant increase in scheduled mental health visits. Project Welcome Home included Intensive Case Management with a low client‐staff ratio led by licensed staff with behavioral health training. Research has shown that experiencing homelessness is one factor that leads to ED visits among psychiatric patients, suggesting an unmet need for mental health care.^5^, ^18^ Our findings suggest that these visits are amenable to prevention by providing housing with associated low‐barriers mental health services. We did not find a significant reduction in other acute medical care visits, although the point estimates for both ED visits and psychiatric admissions were less than one. These results differ from those reported in studies of PSH that used uncontrolled designs. These found large reductions in service use.^12^, ^19^, ^20^ Without controls, these findings are susceptible to regression to the mean.^20^, ^21^ Our finding of decreased use in later span years independent of group assignment suggests regression to the mean. Two related RCTs found statistically significant reductions in ED visits, nonstatistically significant reductions in inpatient medical hospitalizations and increases in psychiatric hospitalizations.^11^, ^22^ People who are high users of services likely have unmet health needs that become apparent once housed. Our results may have underestimated improvements due to misclassification: 14 percent of participants in our study did not engage in housing, although most of these participants did receive case management services. At the time of the study, SCC increased its provision of PSH.^23^ Thirty‐six percent of those in the control group received PSH or other forms of subsidized housing through other programs. This would bias our results toward the null and could have obscured our ability to see subtle differences.

Despite selection criteria that identified those at highest risk for frequent utilization, and thus, most likely to experience mental health and substance use disabilities, we found the 86 percent of individuals randomized to PSH entered housing and remained housed, on average, for 93 percent of the time in the study. Engagement and retention in housing is an important priority for policy makers.^24^ Our selection criteria aimed to identify those who were the highest users of services. Similar to Coordinated Entry, a Housing and Urban Development policy that requires Counties to prioritize those with the most significant barriers to housing to receive housing and homeless assistance services, we designed the triage tool to identify those who, due to their high use of acute care services and jail, likely faced the biggest barriers. Thus, our study has implications for jurisdictions who are using coordinated entry to provide PSH only to those at highest risk. After our initial screening using administrative data, only two individuals refused additional outreach and, after screening in, only one refused to participate in randomization, suggesting that this population is interested in receiving services. We found that by providing housing with appropriate services, the vast majority of high‐risk individuals could be housed successfully. Prior pre–postliterature has suggested that upwards of 85 percent of people engaged in PSH remain housed. We found that 86 percent of high‐risk individuals randomized to the intervention entered housing and these individuals remained housed for the vast majority of the time. This finding extends the finding of pre–poststudies in two ways. While pre–poststudies cannot address the issue of engagement, we found that, even among the highest risk population, the intervention was able to engage 86 percent in housing. Our study's use of a targeting tool include people whose usage patterns suggest that they will have the highest ongoing acute care use provides additional reassurance that even the most high‐risk individuals can be successfully housed using a Housing First approach with intensive case management. The housing patterns we found, however, suggest the need for flexibility. Consistent with the experience of many Housing First programs, over two‐thirds of the housed intervention participants required rehousing after their first placement did not succeed. The ability to offer a new housing placement is a key component of successful Housing First strategies when working with high complexity populations. With the widespread use of Coordinated Entry that will require that counties place individuals with similar risk profiles into PSH, our findings provide support for the need for flexibility, including the ability to rehouse individuals, in order to serve those at highest risk. Our results offer a measured sense of expected changes in their use of other services.

We found a similar high mortality rate in both treatment and control groups. Individuals experiencing homelessness have a greater age‐adjusted mortality rate than housed counterparts.^25^ Among those who died, 89 percent of those in the intervention group had been housed compared with 28 percent in the control group. After longstanding homelessness, housing may not be sufficient to prevent or delay death. However, avoiding deaths while people are homeless has value. The study excluded those with metastatic cancer or those who health care providers deemed eligible for hospice. The high death rate despite these exclusions suggests the vulnerability of the population and the challenge of predicting mortality. It is possible that some of the participants would have benefited from referral to a higher level of care instead of PSH. This requires further evaluation.^26^, ^27^

We found no differences in criminal justice system encounters between participants in the intervention and control groups, which is consistent with prior research.^28^ Individuals experiencing homelessness are more likely to be arrested for offenses that can be directly attributed to the state of being homeless,^29^ including trespassing, sleeping in vehicles, panhandling, and public use of illicit drugs and alcohol. City‐wide bans on public camping and panhandling have increased by 69 and 43 percent, respectively, over the past decade.^30^ The lack of a difference may be attributed to the fact that some of the jail stays experienced by individuals who received housing were caused by outstanding warrants that the criminal justice system served once the individual received housing. For this high‐risk population, programs to help detect and mitigate risk of criminal justice involvement, as well as policies that support alternatives to incarceration, may need to be better integrated into PSH programs. This will require future study.

### 4.1 Limitations

Our study has important limitations. We used a randomized, intention‐to‐treat framework so that all individuals who enrolled in the study were included when evaluating outcomes. Sixteen percent of individuals in the treatment group never received housing, and 36 percent of those in the control group received PSH through other programs during a time of expansion of PSH in Santa Clara.^23^ This could bias our findings toward the null. In addition, our higher‐than‐expected mortality rate among participants limited follow‐ up periods for participants who died. It is possible that we missed deaths among the control group. This would artificially reduce service utilization in this population and bias results toward the null. Only a minority of individuals had a history of criminal justice system interactions in the 2 years prior to enrollment. This may have limited our power to detect differences, although our findings are consistent with prior research. We had access to an integrated database that allowed us to examine use of multiple county services. However, we were unable to detect service use that may have occurred either outside of the county or that occurred in other health care facilities within SCC, with the exception of psychiatric inpatient services. This may have led to underestimation of service use in the study population. If enrollment in the PSH intervention resulted in increased likelihood of referral for medical care to the County hospital (as compared to other hospitals in the County), this may have differentially impacted our ability to detect service use in the intervention group. Alternatively if, due to their housing, participants in the intervention group preferentially increased their use of other hospitals, this could have led us to deflate use in the intervention group.

## 5 CONCLUSIONS

We found PSH delivered in a Housing First method delivering services through an Intensive Case Management model with a low client to staff ratio successfully housed chronically homeless individuals who were high users of multiple public systems of care. While the intervention reduced use of the psychiatric ED and shelters and increased housing, it did not reduce ED use for physical health care or hospitalizations. We found high death rates for participants in both groups, emphasizing the medical frailty of the population. While early, uncontrolled, studies of PSH may have overstated expected reductions in inpatient and ED care, these reductions may be harder to realize in high need populations who experience underuse of services. However, the intervention's ability to house, successfully, a high proportion of the most high‐risk chronically homeless population who were the highest user of multiple systems of care demonstrates the potential of Housing First to house the highest risk individuals.

## Supporting information

Author Matrix

Table S1
