# Supplementary material for: A randomized trial of permanent supportive housing for chronically homeless persons with high use of publicly funded services
Source: Health Serv Res. 2020 Sep 25;55(Suppl 2):797–806. doi: 10.1111/1475-6773.13553 (PMC7518819; doi:10.1111/1475-6773.13553)
Supplement: Supplementary file 2 — Table S1 [file HESR-55-797-s002.docx]

**Appendix Table 1. Logistic and negative binomial regression analysis of treatment status on additional outcome variables**

|  | **Total Bed Days** | **ED Visits Discharged Home** | **ED Visits Admitted** | **ED Visits Delivered By Ambulance** | **ED Visits Delivered By Police** | **Jail Days** | **Shelter Stays** |
| --- | --- | --- | --- | --- | --- | --- | --- |
| Treatment Group | 1.12 | 0.83 | 0.96 | 0.91 | 0.66* | 0.82 | 0.35** |
|  | [0.79,1.59] | [0.64,1.08] | [0.69,1.34] | [0.65,1.25] | [0.44,0.99] | [0.55,1.23] | [0.20,0.61] |
| Span 1 (reference) | - | - | - | - | - | - | - |
| Span 2 | 0.85 | 0.81** | 0.80* | 0.88 | 1.02 | 1.17 | 0.54** |
|  | [0.63,1.15] | [0.69,0.93] | [0.65,0.98] | [0.75,1.04] | [0.78,1.32] | [0.86,1.60] | [0.35,0.83] |
| Span 3 | 0.93 | 0.74** | 0.74* | 0.74* | 0.97 | 1.41* | 0.34** |
|  | [0.66,1.32] | [0.61,0.89] | [0.56,0.97] | [0.59,0.93] | [0.68,1.38] | [1.01,1.96] | [0.21,0.55] |
| Span 4 | 0.71 | 0.64** | 0.57** | 0.66** | 0.87 | 1.37 | 0.29** |
|  | [0.43,1.18] | [0.49,0.84] | [0.39,0.84] | [0.49,0.88] | [0.57,1.34] | [0.91,2.05] | [0.12,0.68] |
| N | 1070 | 1070 | 1070 | 1070 | 1070 | 1070 | 1070 |

Notes: All results presented as incidence rate ratios for all other outcomes from negative binomial regressions. 95% confidence intervals also presented. Covariates include treatment status as well as span indicators to control for the time since enrollment. No other covariates were included as the treatment was randomly assigned.

Abbreviations: ED – emergency department

*p<0.05, **p<0.01
